# Verbal stimuli allow for symmetrical S-R priming effects between size and space

**DOI:** 10.1038/s41598-024-77806-8

**Published:** 2024-11-05

**Authors:** Melanie Richter, Peter Wühr

**Affiliations:** Department of Psychology, TU Dortmund University, Emil-Figge-Straße 50, 44227 Dortmund, Germany

**Keywords:** Psychology, Human behaviour

## Abstract

The spatial-size association of response codes (SSARC) effect refers to the observation that left responses are faster and more accurate to small stimuli whereas right responses are faster and more accurate to large stimuli, as compared to the reverse assignment. The underlying spatial-size associations are strongly asymmetrical with physical size/location stimuli and vocal location/size responses and allow for regular but not reciprocal SSARC effects. Recent evidence, however, points towards an important role of stimulus mode in the emergence of reciprocal compatibility effects. We investigated the reciprocity of the SSARC effect with a different stimulus mode, namely with verbal size/location stimuli and vocal responses. In a size-location task, participants vocally responded to the words “small” or “large” by saying “left” or “right” according to a compatible (“small”-“left”/“large”-“right”) or an incompatible mapping (“small”-“right”/“large”-“left”). In a location-size task, participants vocally responded to the words “left” or “right” by saying “small” or “large” according to a compatible (“left”-“small”/“right”-“large”) or an incompatible (“left”-“large”/“right”-“small”) mapping. We observed a regular and a reciprocal SSARC effect of similar size indicating symmetrical spatial-size associations. While regular SSARC effects thus emerge with verbal and physical size stimuli, reciprocal SSARC effects only emerge with verbal but not with physical location stimuli and vocal responses. Theoretical accounts of the SSARC effect differ in whether they predict reciprocal effects and whether they can account for the effect of stimulus mode on the reciprocal SSARC effect.

**Supplementary Information:**

The online version contains supplementary material available at 10.1038/s41598-024-77806-8.

## Introduction

Certain assignments between stimulus and response alternatives allow for a better performance in terms of response speed and accuracy than other assignments of stimulus and response alternatives^1,2^. This observation is commonly referred to as *Stimulus-Response (S-R) Compatibility* and the performance advantage, which arises for “compatible” compared to “incompatible” mappings, is termed *compatibility effect*. Compatibility effects do not only occur when stimuli and responses vary on the same dimension, such as horizontal location, but also when stimuli and responses vary on different dimensions, such as physical size and spatial location^2,3^. When stimuli and responses vary on different – but associated – dimensions, the question of reciprocity arises: Do tasks in which dimension A (e.g., size) varies on the stimulus side and dimension B (e.g., location) varies on the response side, and tasks in which dimension B varies on the stimulus side and dimension A on the response side produce equivalent compatibility effects or not?

Associations between physical stimulus size and space (i.e., horizontal response location) give rise to the so-called *spatial-size association of response codes (SSARC) effect*^4–6^. The SSARC effect describes the phenomenon that left responses are faster and more accurate to physically small(er) stimuli whereas right responses are faster and more accurate to physically large(r) stimuli. The SSARC effect thus indicates that the mental representations of physical size and space are associated, which can be referred to as *spatial-size associations*. Several studies have so far investigated the SSARC effect and, for example, revealed that the effect also emerges when size is a task-irrelevant stimulus feature, thereby testifying to the automatic processing of physical size and its subsequent association with space^6,7^. Moreover, Wühr et al.^8^ demonstrated that the SSARC effect also occurs with vocal responses and thus seems to be independent of response mode. Spatial-size associations therefore do not seem to rely on direct links between stimulus and response codes but instead seem to be located on an intermediate representational level.

Spatial-size associations robustly emerge in the form of a regular SSARC effect with physical size stimuli and spatial responses. However, they do not seem to emerge in the opposite direction in the form of a reciprocal SSARC effect with spatial stimuli and responses referring to physical size^9^. In a previous study, we investigated the reciprocity of the SSARC effect by comparing the compatibility effects in two vocal choice-response tasks^9^. In a size-location task, participants vocally responded to a physically small (2 cm) or physically large (4 cm) stimulus by saying “left” or “right” according to a compatible (small-“left”; large-“right) or an incompatible mapping (small-“right”; large-“left”). In a location-size task, participants vocally responded to a left or right physical stimulus location by saying “small” or “large” according to a compatible (left-“small”; right-“large”) or an incompatible (left-“large”; right-“small”) mapping. Importantly, we used vocal responses because a) SSARC effects with manual and vocal responses are of comparable size^8^ and b) manual responses referring to physical size are difficult to implement.

The results of our study revealed a significant regular SSARC effect in the size-location task but a non-significant reciprocal SSARC effect in the location-size task when outliers were excluded. Without the exclusion of outliers, merely small reciprocal SSARC effects emerged. These findings provide preliminary evidence that spatial-size associations underlying the SSARC effect with physical stimuli and vocal responses are strongly asymmetrical. While physical stimulus sizes can prime vocal location responses, physical stimulus locations cannot prime vocal size responses with a similar strength^9^.

However, a recent study investigating the reciprocity of the so-called *spatial-numerical associations of response codes (SNARC) effect* has shown that the reciprocity of the SNARC effect depends on the stimulus mode^10^. The regular SNARC effect describes the phenomenon that left responses are faster and more accurate to small(er) numbers whereas right responses are faster and more accurate to large(r) numbers^11–13^. The reciprocal SNARC effect, in turn, describes the phenomenon that responses referring to small(er) numbers are faster and more accurate to left locations whereas responses referring to large(r) numbers are faster and more accurate to right locations. While the regular SNARC effect emerges with numerosity, digit as well as verbal number stimuli and vocal responses, the reciprocal SNARC effect only emerges with verbal location stimuli (i.e., location words “left” and “right”) and vocal responses but not with physical location stimuli (i.e., stimuli appearing in the left and right screen half) and vocal responses. This implies that stimulus mode affects the emergence of the reciprocal but not the regular SNARC effect^10^.

In our previous experiment investigating the reciprocity of the SSARC effect, we did not observe a reciprocal SSARC effect with physical location stimuli and vocal size responses. Since we have found that a reciprocal effect of the related SNARC effect only emerges with verbal but not with physical location stimuli, it seems plausible to assume that a reciprocal SSARC effect might also emerge with verbal instead of physical location stimuli. In our present experiment, we therefore investigate the reciprocity of the SSARC effect with verbal stimuli and vocal responses. In the size-location task, participants vocally respond to the size word “small” or “large” by saying “left” or “right” according to a compatible (“small”-“left”; “large”-“right”) or an incompatible (“small”-“right”; “large”-“left”) mapping. In the location-size task, participants vocally respond to the location word “left” or “right” by saying “small” or “large” according to a compatible (“left”-“small”; “right”-“large”) or an incompatible (“left”-“large”; “right”-“small”) mapping.

In doing so, we aim to address two research questions. Firstly, does the reciprocal SSARC effect emerge with verbal location stimuli instead of physical location stimuli or, in other words, does the reciprocal SSARC effect – similar to the reciprocal SNARC effect – depend on stimulus mode? Since stimulus mode might not only affect the reciprocal SSARC but potentially also the regular SSARC effect, we secondly ask: Does the regular SSARC effect also emerge with verbal instead of physical size stimuli or, in other words, is the regular SSARC effect independent of stimulus mode? While the regular SSARC effect seems to be independent of response mode^8^, its independency of stimulus mode still remains to be tested.

We use the term regular SSARC effect to denote the compatibility effect between physical stimulus size and spatial responses as in a typical SSARC task and we use the term reciprocal SSARC effect to denote a compatibility effect in the opposite direction, that is, between spatial stimuli and responses referring to physical size. Importantly, several theoretical accounts which have been proposed to explain the SSARC effect differ in whether they predict bidirectional associations between size and space and whether they predict (or at least can account for) an influence of stimulus mode on the emergence of reciprocal and/or regular SSARC effects.

The *polarity correspondence principle* by Proctor and colleagues^14^ assumes that in many binary classification tasks, one stimulus and response alternative is assigned a positive polarity whereas the other stimulus and response alternative is assigned a negative polarity. Corresponding polarities of stimuli and responses in turn lead to faster and more accurate responses than non-corresponding polarities^14–16^. According to the polarity correspondence principle, the SSARC effect arises because the stimulus and response alternatives “small” and “left” are given negative polarity while the stimulus and response alternatives “large” and “right” are given positive polarity. In our view, the polarity correspondence principle predicts bidirectional and symmetrical SSARC effects since “small”/“left” and “large”/“right” are encoded as negative and positive polarity regardless of being varied as a stimulus or response feature. Moreover, stimulus mode should neither influence the emergence of the regular nor the reciprocal SSARC effect since polarities should be assigned to the bipolar dimensions of space and size regardless of their given format.

The *working memory (WM) account* by van Dijck and colleagues^17,18^ proposes that it is the serial order in which stimuli of a given task are stored in WM that corresponds with spatial response location thus leading to compatibility effects. While stimuli at early serial positions are associated with left responses, stimuli at late serial positions are associated with right responses^17,18^. In terms of the WM account, in a typical SSARC task, stimulus sizes are spontaneously stored in an ascending order in WM. Accordingly, small stimuli then facilitate left responses while large stimuli facilitate right responses. Even when extending the WM account by the *mental whiteboard hypothesis*^19–21^, an account which proposes that the serial order of stimuli is encoded in (verbal) WM by connecting the stimuli to spatial position markers, the possibility of reciprocal effects is not addressed. To explain a reciprocal SSARC effect, one would have to assume that location stimuli are serially stored by connecting them to spatial position markers. Those spatial position markers could, however, only prime size-related responses if responses were also spontaneously stored in a canonical order and connected to spatial position markers. The predictions of the WM account in terms of reciprocity are thus unclear. The fact that the serial order of stimuli is encoded in verbal WM allows for a regular SSARC effect with verbal instead of physical size stimuli but also for a reciprocal SSARC effect with verbal instead of physical location stimuli.

The *correlations in experience (CORE) principle* by Pitt and Casasanto^22^ assumes that associations between the mental representations of two dimensions arise because those dimensions are correlated in people’s experience, that is, in their natural or cultural world, in the first place. Wühr et al.^8^ applied the CORE principle to account for the SSARC effect and proposed that people’s grasping habits determine the associations between physical size and space: While people tend to grasp smaller and lighter objects with their weaker non-dominant hand, they tend to grasp larger and heavier objects with their stronger dominant hand. Even though during grasping movements, size (i.e., object size, grip aperture) and space (i.e., object and hand location) are consistently involved as stimulus and response features, the decision between effectors (left or right) is situated only on the response level. It remains ambiguous if grasping habits should consequently predict unidirectional or bidirectional SSARC effects. However, given that “any experience should affect metaphorical mappings between any two conceptual domains”^22^, CORE can in principle account for any kind of (lacking) mapping effect assuming that this relationship has (not) previously been experienced. Moreover, the broader *hierarchical mental metaphors theory* (HMMT;^23,24^) explicitly allows for the co-existence of different correlations involving the same dimensions. The CORE principle and the HMMT thus fail to make clear predictions regarding the bidirectionality and symmetry of the SSARC effect. The CORE principle can, however, explain regular and reciprocal SSARC effects with verbal stimuli and vocal responses if one assumes that spatial-size associations are not restricted to the stimulus or response mode, the correlation was originally experienced in. This assumption is in line with HMMT^23,24^ and previous findings that have demonstrated that spatial-size associations are not limited to the manual response mode^8^.

## Methods

The experiment was preregistered on the website OpenScienceFramework (OSF) (https://osf.io/uqvz2).

### Participants

In our previous study^9^, in which we investigated the reciprocity of the SSARC effect with physical location/size stimuli and vocal size/location responses, we observed a strong main effect of mapping (ɳ^2^_p_ = .22), and a strong two-way interaction between task and mapping (ɳ^2^_p_ = .16). Accordingly, we assumed a ɳ^2^_p_ of 0.20 for the main effect of mapping and for the two-way interaction for the present experiment. We conducted a power analysis with the software MorePower^25^ which revealed that a sample size of 54 participants is sufficient to detect an effect of this size with high power (1-beta = 0.95) at the standard 0.05 alpha error probability. To account for the exclusion of potential outlier data sets, we planned to test 60 participants.

Sixty volunteer students (47 female, 13 male) with a mean age of 22.333 years (*SD* = 3.139) took part in our experiment. They were compensated by either course credit or a payment of 10 Euro. According to self-report, all participants had normal (*N* = 35) or corrected-to-normal (*N* = 25) vision. Fifty-four participants classified themselves as right-handed, whereas six participants classified themselves as left-handed. Even though a prior study has shown that handedness affects the SSARC effect^8^, we decided to include left-handed participants in our sample (i.e., prior to data collection) because they typically merely show a weaker but no reverse SSARC effect. All participants gave their informed consent prior to participation. Moreover, the local Ethics Committee at TU Dortmund University approved the experimental protocol for the present study (GEKTUDO_2022_36). We confirm that all methods were performed in accordance with the relevant guidelines and regulations.

### Apparatus and stimuli

Participants sat in front of a 24-inch color monitor with a viewing distance of approximately 50 cm. We employed the software EPrime 3.0 (Psychology Software Tools; Sharpsburg, PA, USA) to control stimuli presentation, register vocal responses, and measure reaction time (RT). A small plus sign (Courier font, size 18 pt) was presented at the screen center at the beginning of each trial and thus served as a fixation point. All imperative stimuli were presented at the center of the screen. Moreover, all imperative stimuli were written in 40 pt in Times New Roman and presented in black on a white background. In the size-location task, the size words “small” and “large” served as imperative stimuli to which participants responded vocally by saying “left” or “right” into a microphone. The microphone was placed in front of the participants and centrally aligned to their body midline. It was connected to the voice-key of the Chronos console (Psychology Software Tools; Sharpsburg, PA, USA) to register RTs and record participants’ vocal responses. Each vocal response was stored in a separate sound file and later checked for accuracy. In the location-size task, the location words “left” and “right” served as imperative stimuli to which participants responded vocally by saying “small” or “large”. Even though all stimuli were presented in German and participants responded in German, we use the corresponding English words in the text for consistency. Please note that we differentiate between verbal stimuli (i.e., in the sense of written) and vocal responses (i.e., in the sense of spoken). In other words, all stimuli were presented visually in the form of size or location words whereas all responses were given vocally.

### Procedure

**Fig. 1:**
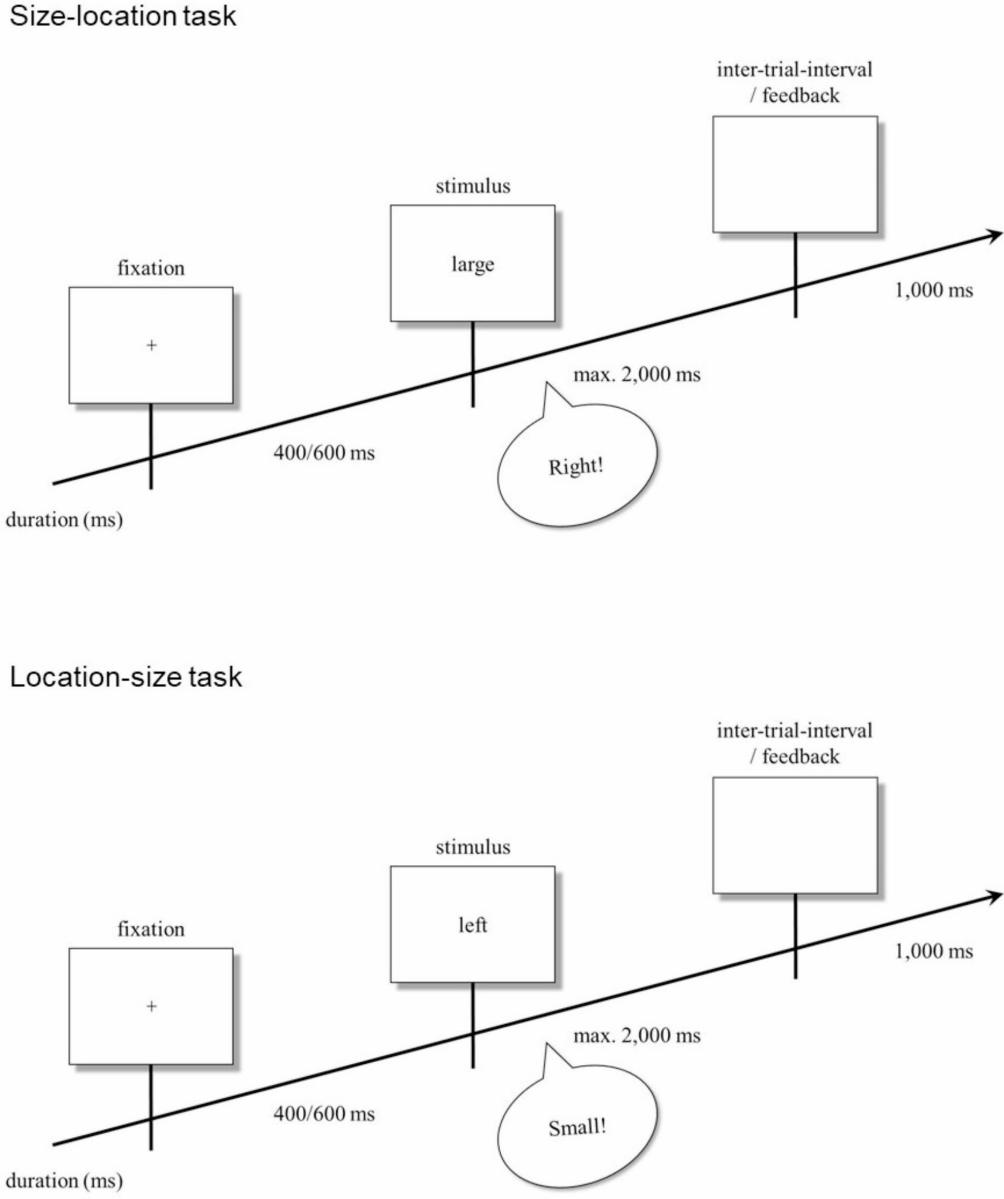
Time course of events in a typical trial of the size-location task (upper panel), and the location-size task (lower panel) according to compatible mappings. Stimulus displays are depicted as rectangles whereas a vocal response is depicted as a speech bubble.

Four conditions resulted from the orthogonal combination of two tasks (size-location task, location-size task) and two S-R mappings (compatible, incompatible) and were thus completed by each participant. In the size-location task, participants responded vocally to the stimulus words “small” or “large” by saying “left” or “right” according to a compatible mapping (“small”-“left”, “large”-“right”) or an incompatible mapping (“small”-“right”, “large”-“left”). In the location-size task, participants responded vocally to the stimulus words “left” or “right” by saying “small” or “large” according to a compatible mapping (“left”-“small”, “right”-“large”) or an incompatible mapping (“left”-“large”, “right”-“small”). The time course and sample stimuli of the size-location task and the location-size task are depicted in Fig. 1.

At the beginning of each condition, instructions informed participants about the content and the procedure of the task. Each condition contained one training block with 10 trials and two experimental blocks with 40 trials each. Trials were randomized within each block. At the beginning of each trial, a fixation point was presented for 400 or 600 ms, with both durations occurring equally often in each block. The imperative stimulus was then presented for a maximum of 2,000 ms or until a response was given. An empty screen was presented for 1,000 ms during an inter-trial interval after a response was recorded, whereas a corresponding error message was presented during the inter-trial interval after a missing response. Since the program could not determine the accuracy of the vocal responses, participants did not receive feedback about the correctness of their responses. Instructions were repeated at the beginning of each experimental block to remind participants of the present task and S-R mapping. Between blocks participants were allowed to take a break or to continue with the subsequent block.

Both variables (i.e., task and S-R mapping) were varied within-subjects but between different blocks of trials. The order of tasks (size-location or location-size task first) and the order of mappings (compatible or incompatible mapping first) were both counterbalanced between participants. Participants completed both S-R mapping conditions consecutively within one task and the order of mappings was consistent between tasks for one participant. The experiment took about 30 min. The experimenter stayed in the laboratory for the practice trials but left the room before participants started the experimental blocks.

### Design and data analysis

Before the analysis, the audio files with participants’ vocal responses were checked in terms of accuracy. Errors were manually entered into the data file. The experimental design was a two-factorial (*Task* x *Mapping*) within-subjects design. The factor *Task* had two levels: the size-location task and the location-size task. The factor *S-R Mapping* also had two levels: the compatible mapping and the incompatible mapping. We employed RTs of correct vocal responses and error percentages as dependent variables. For consistency reasons, the onset of participants’ vocal responses was used both to measure RTs and determine accuracy. A response was thus considered erroneous when the first sound could be attributed to the incorrect response.

We planned to investigate the impact of the two independent variables (i.e., Task, Mapping) on the dependent variables (i.e., RTs, error percentages) with a two-way ANOVA. In case of a significant two-way interaction, we planned to employ *t* tests to determine the source of the interaction. Error percentages often contain a large number of ties that are often excluded from non-parametric tests, which biases the results towards H1. We therefore planned to employ *t* tests instead of non-parametric tests even though error percentages typically violate the normality assumption. Moreover, we additionally report the Bayes Factor (BF) for each pairwise comparison because we intended to evaluate the evidence for both the null (absence of effect) and the experimental (presence of effect) hypothesis^26^. To interpret the BF values, we follow Jeffreys’ (1961) evidence categories (as cited in Lee and Wagenmakers^27^). Since overall outliers might be driving reciprocal SSARC effects^9^, we conducted the same analyses once again after the exclusion of outlier participants according to the Tukey criterion^28^. We report these results in the **Supplementary Information**.

Previous studies have shown that response speed (i.e., RT) affects the size of compatibility or congruency effects^29^. The regular SSARC effect with physical stimuli and manual or vocal responses has been shown to increase with increasing RTs^9,30^. Moreover, small trends of reciprocal SSARC effects with physical stimuli and vocal responses merely emerged for higher RT levels and also increased with increasing RTs^9^. Since we employed verbal stimuli instead of physical stimuli in the present experiment, we conducted a distributional analysis (cf. **Supplementary Information**) to determine the time course of the regular as well as the reciprocal SSARC effect with verbal stimuli and vocal responses. Moreover, with the distributional analysis we also aimed to detect small reciprocal SSARC effects, which might have emerged for specific RT levels but did not reach significance in the omnibus analysis. To analyze the time course of the mapping effects, we employed Ratcliff’s method of vincentizing^31^. We rank-ordered RTs for each participant and condition before we divided them into four quartiles and computed the corresponding means. The means were then subjected to a three-factorial ANOVA with *Task* (size-location task, location-size task), *Mapping* (compatible, incompatible) and *Quartile* (1–4) as within-subject variables.

## Results

### Data trimming

On an overall level, we excluded one participant (number 44 in the dataset) whose mean error percentage exceeded 20% in one of the two tasks. After excluding this dataset, the highest error percentage was 16.88% in the size-location task, and 14.81% in the location-size task. Our final sample thus contained 59 participants. On a trial level, we excluded trials with RTs below 100 ms or above 1,500 ms and the first trial in each block. Participants’ responses were too fast (i.e., RT < 100 ms) in less than 1% of trials in both the size-location (*M* = 0.21%, *SD* = 0.84) and location-size task (*M* = 0.09%, *SD* = 0.33). Similarly, participants’ responses were too slow (i.e., RT > 1,500 ms) in less than 1% of trials in both the size-location (*M* = 0.29%, *SD* = 0.66) and location-size task (*M* = 0.41%, *SD* = 0.89).

### Reaction times (RTs)

The two-factorial ANOVA with *Task* and *Mapping* as within-subject factors revealed two significant main effects. The significant main effect of *Task*, *F*(1, 58) = 15.99, *MSE* = 2,730.71, *p* < .001, ɳ^2^_p_ = .22, indicated shorter RTs in the size-location task (*M* = 508 ms, *SD* = 76) than in the location-size task (*M* = 536 ms, *SD* = 98). The significant main effect of *Mapping*, *F*(1, 58) = 62.60, *MSE* = 2,628.03, *p* < .001, ɳ^2^_p_ = .52, reflected shorter RTs with the compatible mapping (*M* = 496 ms, *SD* = 76) than with the incompatible mapping (*M* = 548 ms, *SD* = 92). Importantly, however, the two-way interaction was non-significant, *F*(1, 58) = 2.05, *MSE* = 1,176.87, *p* = .157, ɳ^2^_p_ = .03, revealing similar mapping effects in the two tasks.

**Fig. 2:**
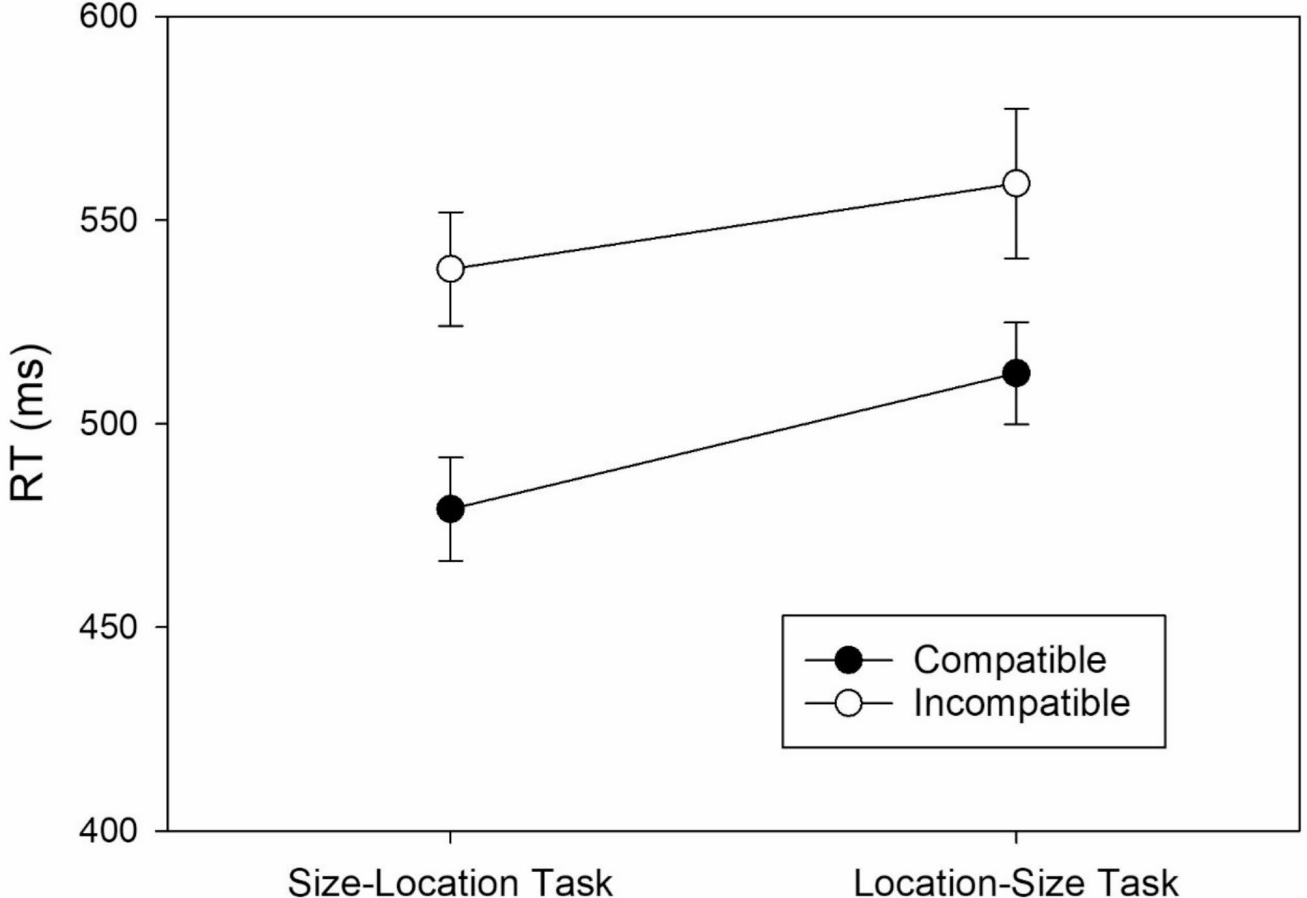
RTs of correct responses as a function of Task and S-R Mapping. Error bars reflect 95% confidence intervals for within-subjects designs^32^.

For each task, we conducted a pairwise comparison between the compatible and incompatible mapping to determine the size of the mapping effect. In the size-location task, RTs were significantly shorter with the compatible than with the incompatible mapping, *t*(58) = 8.67, *p* < .001, *d* = 1.13, BF_+0_ > 10,000.00, reflecting a regular SSARC effect of 59 ms (cf. Figure 2) and extreme evidence for its presence. In the location-size task, RTs were significantly shorter with the compatible than with the incompatible mapping, *t*(58) = 5.12, *p* < .001, *d* = 0.67, BF_+0_ = 4,650.53, revealing a reciprocal SSARC effect of 46 ms (cf. Figure 2) and extreme evidence for its presence.

### Error percentages

The two-factorial ANOVA with *Task* and *Mapping* as within-subjects factors revealed a significant main effect of *Mapping* and a significant two-way interaction. The significant main effect of *Mapping*, *F*(1, 58) = 37.98, *MSE* = 12.46, *p* < .001, ɳ^2^_p_ = .40, revealed less errors with the compatible mapping (*M* = 2.04, *SD* = 3.34) than with the incompatible mapping (*M* = 4.88, *SD* = 4.62). A non-significant main effect of *Task*, *F*(1, 58) = 0.17, *MSE* = 8.04, *p* = .685, ɳ^2^_p_ < .01, indicated similar error percentages in the size-location (*M* = 3.39, *SD* = 4.19) and the location-size task (*M* = 3.54, *SD* = 4.35). Importantly, however, the significant two-way interaction, *F*(1, 58) = 4.22, *MSE* = 6.94, *p* = .044, ɳ^2^_p_ = .07, revealed different mapping effects in the two tasks.

**Fig. 3:**
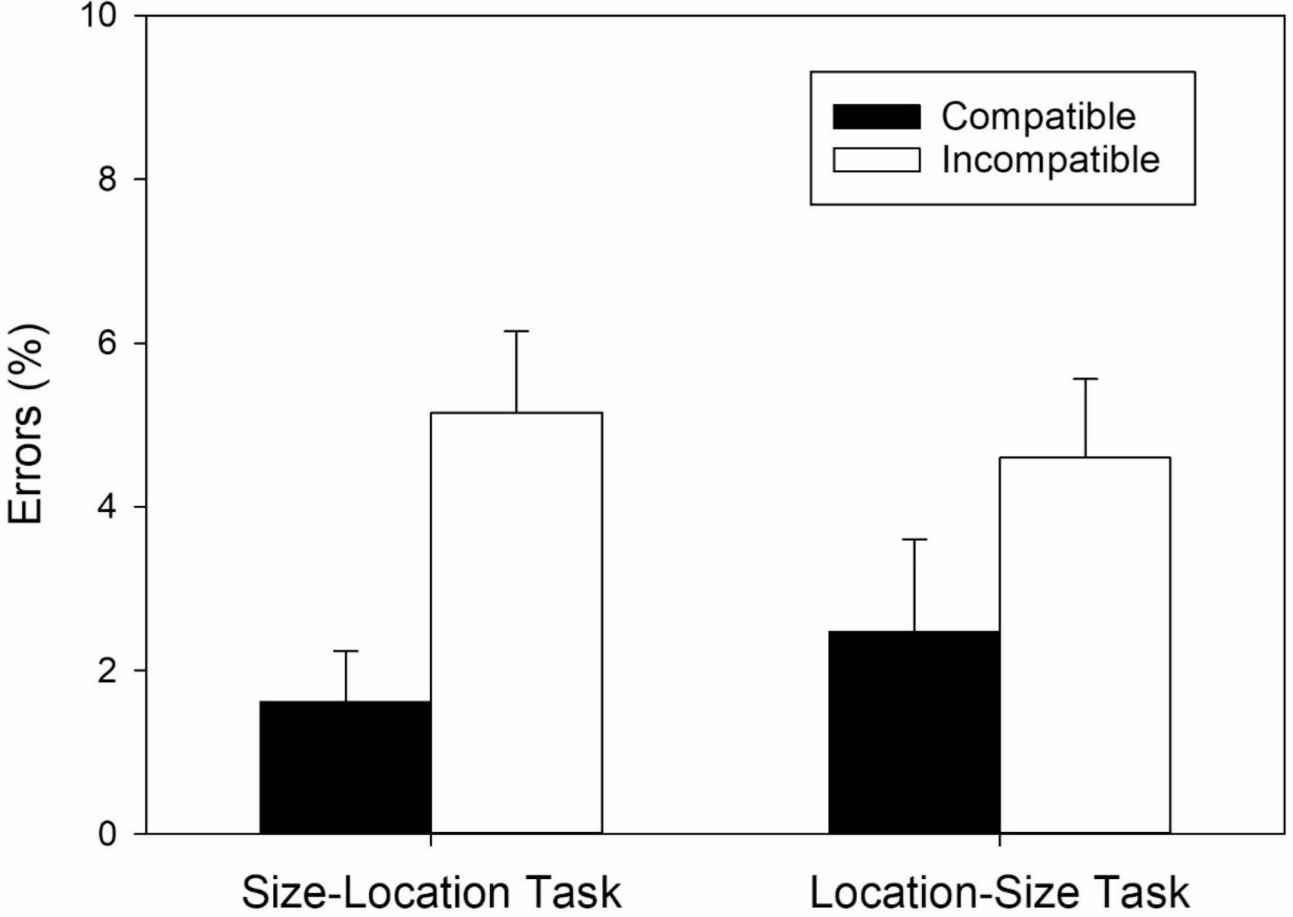
Error percentages as a function of Task and S-R Mapping. Error bars reflect 95% confidence intervals for within-subjects designs^32^.

To determine the source of the two-way interaction, we conducted a pairwise comparison between the compatible and incompatible mapping for each task. In the size-location task, errors were significantly less frequent with the compatible than with the incompatible mapping, *t*(58) = 7.49, *p* < .001, *d* = 0.98, BF_+0_ > 10,000.00, revealing a regular SSARC effect of 3.54% (cf. Figure 3) and extreme evidence for its presence. Likewise, in the location-size task, errors were also significantly less frequent with the compatible than with the incompatible mapping, *t*(58) = 3.23, *p* = .002, *d* = 0.42, BF_+0_ = 14.08, revealing a reciprocal SSARC effect of 2.13% (cf. Figure 3) and strong evidence for its presence.

### Comparison of mapping effects between experiments

We conducted additional analyses to investigate how the stimulus mode affected the regular and the reciprocal SSARC effect by comparing the mapping effects of our previous experiment^9^, where we had used physical size/location stimuli, to the mapping effects of our present experiment, where we used verbal size/location stimuli. We conducted a two-factorial ANOVA with *Mapping* as within-subjects variable, *Experiment* as between-subjects variable and RTs from trials with correct responses as dependent variable for each task separately. For the typical SSARC task (i.e., size-location task), the main effect of *Experiment* was significant, *F*(1, 113) = 9.20, *MSE* = 9,526.33, *p* = .003, ɳ^2^_p_ = .08, indicating higher RTs in the present (*M* = 508 ms, *SD* = 76) than in the previous experiment (*M* = 469 ms, *SD* = 77). The two-way interaction was also significant, *F*(1, 113) = 11.51, *MSE* = 1,240.46, *p* < .001, ɳ^2^_p_ = .09, indicating a larger regular SSARC effect in the present than in the previous experiment. For the reciprocal SSARC task (i.e., location-size task), we observed a similar pattern: the main effect of *Experiment* was significant, *F*(1, 113) = 67.09, *MSE* = 13,541.43, *p* < .001, ɳ^2^_p_ = .37, indicating higher RTs in the present (*M* = 536 ms, *SD* = 98) than in the previous experiment (*M* = 410 ms, *SD* = 76). The two-way interaction was also significant, *F*(1, 113) = 17.20, *MSE* = 1,538.58, *p* < .001, ɳ^2^_p_ = .13, indicating a larger reciprocal SSARC effect in the present than in the previous experiment.

## Discussion

In our present experiment, we investigated the reciprocity of the SSARC effect with verbal stimuli and vocal responses by comparing the compatibility effects in a typical size-location task and a reciprocal location-size task. In particular, we aimed to find out if the regular SSARC effect emerges with verbal instead of physical size stimuli and if the reciprocal SSARC effect emerges with verbal instead of physical location stimuli. For both RTs and error percentages, we observed a regular SSARC effect with verbal size stimuli and vocal location responses: Participants were faster and more accurate when saying “left” to the size word “small” and when saying “right” to the size word “large” as compared to the opposite mapping. While the regular SSARC effect typically emerges with physical sizes as stimuli^6^, the present results demonstrate that the effect also emerges with size words as stimuli.

A direct comparison between the results with physical^9^ and verbal stimuli, however, reveals that stimulus mode affects the effect size: The regular SSARC effect was larger with verbal instead of physical stimuli. Yet, this finding can be attributed to the finding of overall higher RTs with verbal stimuli and the observation that the regular SSARC effect increased with increasing RTs (cf. **Supplementary Information**), which is also in line with previous observations^9,30^. The regular SSARC effect thus not only occurs with different response modes (manual, vocal;^8^) but also with different stimulus modes (physical, verbal;^9^).

Most interestingly, for both RTs and error percentages we also observed a reciprocal SSARC effect with verbal location stimuli and vocal size responses: Participants were faster and more accurate when saying “small” to the location word “left” and when saying “large” to the location word “right” as compared to the opposite mapping. Moreover, for RTs the reciprocal and the regular SSARC effect were of similar size indicating bidirectional and symmetrical spatial-size associations. Location words can thus influence the selection and execution of vocal size responses to a similar extent as size words can influence the selection and execution of vocal location responses. For error percentages, the regular SSARC effect was larger than the reciprocal SSARC effect indicating bidirectional but asymmetrical spatial-size associations. The distributional analysis (cf. **Supplementary Information**) further demonstrated that the time course of the reciprocal SSARC effect was similar to the time course of the regular SSARC effect: the compatibility effect increased with increasing RTs.

The observation that the reciprocal SSARC effect emerges with verbal location stimuli and vocal size responses is of particular interest because it does not emerge with physical location stimuli and vocal size responses^9^. In our previous study with the same experimental design, we had employed physical location stimuli and vocal size responses and observed a non-significant reciprocal SSARC effect in the location-size task^9^. The reciprocal SSARC effect therefore depends on stimulus mode: Verbal locations but not physical locations can influence the selection and execution of vocal size responses. Most interestingly, this property seems to be shared with the SNARC effect. In a previous study investigating the reciprocity of the SNARC effect, the reciprocal SNARC effect only emerged with verbal location stimuli and vocal (number) responses but not with physical location stimuli and vocal (number) responses^10^. This consistent finding across both effects indicates that physical location stimuli differ from verbal location stimuli in one (or more) aspect(s) that is (are) essential in eliciting a reciprocal SSARC/SNARC effect.

## Theoretical implications

The present findings have several implications for the theoretical accounts of the SSARC effect, which need to explain the effect of stimulus mode on the emergence of a reciprocal SSARC effect. The polarity correspondence principle assumes that the SSARC effect arises because “small” and “left” are given negative polarity while “large” and “right” are given positive polarity and a correspondence in polarities facilitates performance^14–16^. In our view, this account predicts bidirectional and symmetrical SSARC effects since the binary categories are assigned negative and positive polarity regardless of being situated on the stimulus or response level. This prediction is in line with the bidirectional and symmetrical SSARC effects we observed with verbal location/size stimuli and vocal size/location responses. Yet, in order to also account for the effect of stimulus mode on the emergence of the reciprocal SSARC effect and in particular for the absence of a reciprocal SSARC effect with physical location stimuli and vocal size responses^9^, the polarity correspondence principle would have to claim that physical location stimuli or vocal size responses are not encoded in terms of polarity. However, while previous studies have already shown polarity coding of physical location stimuli^33–36^, the reciprocal SSARC effect in our present experiment demonstrates polarity coding of vocal size responses. The polarity correspondence principle therefore cannot account for the effect of stimulus mode on the emergence of the reciprocal SSARC effect. The polarity correspondence principle has been postulated as a general principle to account for compatibility effects in any binary choice response task in which stimuli and responses vary on bipolar dimensions^14–16^ thus also including both the SSARC and SNARC effect. The consistency between the patterns of reciprocity we observed for the SSARC and SNARC effect is therefore in line with the polarity correspondence principle which assumes one shared mechanism underlying both effects.

The WM account assumes that the SSARC effect arises because stimulus sizes of a given task are spontaneously stored in an ascending order in WM and the early (late) serial position of a small (large) size corresponds with the left (right) response location^17,18^. The mental whiteboard hypothesis^19–21^ further accounts for the encoding of the serial order by proposing that spatial position markers are connected to the stimuli stored in WM. To account for the reciprocal SSARC we observed in our experiment, one would have to assume that not only location stimuli but also size-related responses are serially stored in WM and connected to spatial position markers, which then correspond or do not correspond. The WM account, however, remains mute about such processes. Yet, the observation of a reciprocal SSARC effect with verbal but not with physical location stimuli might be in line with the WM account and attributed to the account’s assumption that the serial order of stimuli is encoded in verbal WM^20^. Nevertheless, the account faces difficulties in explaining why stimulus mode in contrast does not affect the emergence of the regular SSARC effect, which occurs with both verbal and physical size stimuli^9^. The WM account assumes that the same mechanism, that is, the correspondence between the serial position of an item stored in verbal WM and a spatial position, is responsible for the occurrence of different compatibility effects provided that they involve one ordinal dimension and space^17,18^. Since the SSARC and SNARC effect should thus share the same underlying mechanism, the WM account is in line with the consistent patterns we observed for both effects.

The CORE principle assumes that the SSARC effect emerges because people experience correlations between stimulus size and response location in their natural or cultural environment^22^. In line, Wühr et al.^8^ proposed that the people’s habit to grasp larger (heavier) objects with their stronger dominant hand, and smaller (lighter) objects with their weaker non-dominant hand, shapes associations between size and space. Since grasping movements involve size and space (i.e., location) both as stimulus and as response features, but the decision between effectors (left or right) is situated only on the response level, it remains ambiguous if the account accordingly predicts unidirectional or bidirectional SSARC effects. Furthermore, the fact that any kind of experience in which size and space are correlated can give rise to a corresponding mapping effect makes it difficult to derive any clear predictions. Conversely, the observed existence of a reciprocal SSARC effect with verbal stimuli and vocal responses points towards a spatial-size association that was caused by an experience in which people respond to the location word “left” (“right”) with the size word “small” (“large”). In line with Wühr et al.^8^ “other variables, beyond handedness and effector strength, also contribute to the origin and/or the size of SSARC effect” (p. 12) and thus might be responsible for the occurrence of a reciprocal SSARC effect with verbal stimuli. In contrast, our previous observation that a similar reciprocal SSARC effect does not emerge with physical location stimuli and vocal size responses indicates that people do not experience a correlation between physical location stimuli and vocal size responses. The emergence of a regular SSARC effect regardless of stimulus and response format^6,8,9^ meanwhile indicates that either separate experiences for each stimulus format have led to similar regular SSARC effects or that the regular SSARC effect has generalized across different stimulus and response formats which is in line with HMMT^23,24^ and previous evidence^8^. In the latter case, however, it remains unclear, why only the regular but not the reciprocal SSARC effect should generalize across different stimulus formats.

According to the CORE principle, both the SSARC and SNARC effect arise because people experience correlations between physical/numerical size and space in their natural or cultural world^8,22^. The SSARC effect is assumed to result from grasping habits^8^, which do not allow for clear predictions about the bidirectionality and symmetry of the SSARC effect. In contrast, the SNARC effect is assumed to result from finger counting habits^22^, which involve the simultaneous variation of number and space on both stimulus and response level and should thus lead to symmetrical number-space associations. The consistency between the patterns of reciprocity which we observed for the SSARC and SNARC effect is striking given that specific correlational experiences should underlie those compatibility effects. However, the consistency does not falsify the CORE principle assuming that both effects may by chance exhibit similar patterns.

## Potential determinants for the emergence of a reciprocal SSARC effect

Physical location stimuli seem to differ from verbal location stimuli in some kind of property that seems to be essential in eliciting a reciprocal SSARC (and reciprocal SNARC) effect. One potential property – and thus one potential explanation for the influence of stimulus mode on the reciprocal SSARC effect – might be the overall higher RT level with verbal (*M* = 536 ms) than with physical stimuli (*M* = 410 ms). Since the reciprocal SSARC effect increased with increasing RTs, the occurrence of a reciprocal SSARC effect with verbal size stimuli might be mediated by higher RTs. Another potential explanation for the occurrence of a reciprocal SSARC effect with verbal location stimuli might reside in a higher set-level compatibility^37^ with verbal stimuli and vocal responses than with physical stimuli and vocal responses. Even though a higher set-level compatibility could also explain the larger regular SSARC effect with verbal stimuli and vocal responses, the overall higher RT level with this combination of stimuli and responses contradicts this hypothesis. Attention allocation might constitute a third potential explanation: While attention remains focused on the screen center with verbal location stimuli, it is shifted to the left or right with physical location stimuli. Future research could address the question why a reciprocal SSARC effect emerges with verbal but not with physical location stimuli.

An alternative explanation for the occurrence of a reciprocal SSARC effect with verbal stimuli and vocal responses resides within the higher perceptual overlap between the stimuli and responses of the compatible compared to the incompatible mapping condition. Since the German words “klein” (small) and “links” (left) / “groß” (large) and “rechts” (right) have more letters in common than their opposite assignment (“klein” – “rechts”/ ”groß” – “links”), this perceptual overlap might be driving the regular and reciprocal SSARC effect. Importantly, the perceptual overlap should contribute to the regular and the reciprocal effect to the same extent. However, the regular SSARC effect also emerges without perceptual overlap^9^ implying that structural overlap is sufficient to elicit the regular SSARC effect^37^. The observed increase from the regular SSARC effect without perceptual overlap to the regular SSARC effect with perceptual overlap could be attributed to a contribution of perceptual overlap in addition to the structural overlap (but, alternatively, also to a higher overall RT level). It could be argued that (a) the reciprocal SSARC effect, in contrast to the regular SSARC effect, is solely driven by perceptual overlap to the same extent as the regular SSARC effect is driven by structural and perceptual overlap or that (b) both the regular and reciprocal SSARC effect solely depend on perceptual overlap in case it exists – without the involvement of any structural overlap. Both possibilities, however, do not seem very plausible. Most crucially, however, the occurrence of a reciprocal *SNARC* effect with verbal but not with physical location stimuli, which we observed in our previous study^10^, cannot be attributed to perceptual overlap. With regards to perceptual overlap, the compatible mapping in this task (“eins” (one) – “links” / “neun” (nine) – “rechts”) differed from the incompatible mapping (“eins” – “rechts”/”neun” – “links”) only in one single letter, which cannot likely be responsible for the occurrence of the reciprocal effect. Due to the similarity between the SNARC and the SSARC effect, this strengthens the assumption that other factors, besides perceptual overlap, are responsible for the occurrence of reciprocal SNARC and SSARC effects.

## Conclusion

The results of our experiment revealed a regular and a reciprocal SSARC effect of similar size with verbal size/location stimuli and vocal location/size responses. With this combination of stimuli and responses, the spatial-size associations underlying the SSARC effect are thus bidirectional and result in symmetrical effects. Together with a previous study^9^, the results furthermore demonstrate an effect of stimulus mode on the emergence of the reciprocal but not on the emergence of the regular SSARC effect: While the regular SSARC effect occurs both with verbal and physical size stimuli, the reciprocal SSARC effect only emerges with verbal but not with physical location stimuli and vocal responses. This pattern has likewise been observed for the SNARC effect^10^ pointing towards some essential property of verbal location stimuli in the emergence of reciprocal effects. Moreover, these findings have implications for the theoretical accounts of the SSARC effect which, firstly, need to explain the occurrence of a reciprocal SSARC effect and, secondly, need to account for the effect of stimulus mode on the emergence of the reciprocal but not on the emergence of the regular SSARC effect.

## Electronic Supplementary Material

Below is the link to the electronic supplementary material.

Supplementary Material 1

## Data Availability

The dataset has been published on the “Mendeley Data” repository (10.17632/f47vcpbnz2.1). The audio files containing participants’ vocal responses can be obtained by contacting the corresponding author (melanie2.richter@tu-dortmund.de). Materials and codes used in this study can also be obtained by contacting the corresponding author (melanie2.richter@tu-dortmund.de).
