# Supplementary material for: Verbal stimuli allow for symmetrical S-R priming effects between size and space
Source: Sci Rep. 2024 Nov 5;14:26764. doi: 10.1038/s41598-024-77806-8 (PMC11538547; doi:10.1038/s41598-024-77806-8)
Supplement: Supplementary file 1 — Supplementary Material 1 [file 41598_2024_77806_MOESM1_ESM.docx]

**Supplementary Information**

**Distributional analysis for RTs**. We conducted a three-factorial ANOVA with *Task* (size-location task, location-size task), *Mapping* (compatible, incompatible) and *Quartile* (1-4) as within-subject variables and RT means as the dependent variable. Please note that we will only report results involving the variable *Quartile*. The corresponding means are depicted in **Figure S1**.


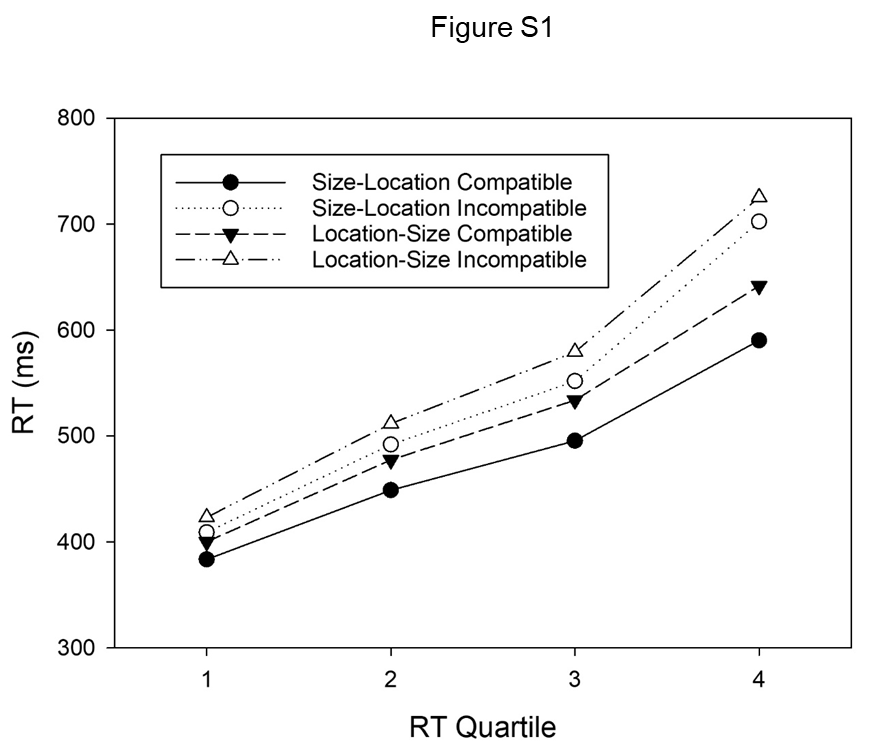


Figure S1: RTs of correct responses as a function of Task, S-R Mapping, and RT Quartile.

Both two-way interactions *Quartile* x *Task* and *Quartile* x *Mapping* were significant. The significant *Task* × *Quartile* interaction, *F*(3, 174) = 8.53, *MSE* = 651.44, *p* < .001, ɳ²_p_ = .13, reflected that, with increasing RTs, mean RTs in the location-size task became increasingly slower compared to the size-location task. The range of RTs was thus smaller in the size-location task compared to the location-size task. The significant *Mapping* × *Quartile* interaction, *F*(3, 174) = 45.53, *MSE* = 1,312.93, *p* < .001, ɳ²_p_ = .44, reflected that mapping effects increased with increasing RTs. Crucially, the three-way interaction, *F*(3, 174) = 2.33, *MSE* = 788.45, *p* = .076, ɳ²_p_ = .04, was non-significant revealing similar time courses of the mapping effects between both tasks.

In the size-location task, the regular SSARC effect increased from 26 ms in the first quartile to 43 ms in the second quartile, 56 ms in the third quartile and 112 ms in the fourth quartile. Post-hoc tests comparing the compatible and incompatible mapping condition for each quartile revealed that the regular SSARC effect was significant in all four quartiles, all *t*s ≥ 5.46, all *p*s*_Tukey_* < .001. In the location-size task, the reciprocal SSARC effect increased from 23 ms in the first quartile to 34 ms in the second quartile, 45 ms in the third quartile and 83 ms in the fourth quartile. Post-hoc tests indicated that the reciprocal SSARC effect was significant in all four quartiles, all *t*s ≥ 4.03, all *p*s*_Tukey_* ≤ .014.

**Exclusion of outliers.** Our previous study on the reciprocity of SSARC effects has shown that overall outliers might be driving reciprocal effects [9]. We thus decided to conduct the same set of analyses after having excluded outlier participants according to the Tukey criterion [28]. The Tukey criterion classifies observations below *Q_25_* – 1,5**IQR* or above *Q_75_* + 1,5**IQR* as outliers. We collapsed data across the mapping variable, applied the criterion to the remaining four variables (mean RT and error percentage in the size-location and location-size task, respectively) and excluded seven further participants (numbers 10, 20, 30, 36, 51, 54 and 60). The remaining sample therefore contained 52 participants.

The exclusion of outliers did not affect the pattern of results in the analysis of RTs. The two-factorial ANOVA with *Task* and *Mapping* as within-subject factors revealed two significant main effects. The significant main effect of *Task*, *F*(1, 51) = 17.43, *MSE* = 2,354.01, *p* < .001, ɳ²_p_ = .25, indicated shorter RTs in the size-location task (*M* = 502 ms, *SD* = 69) than in the location-size task (*M* = 530 ms, *SD* = 93). The significant main effect of *Mapping*, *F*(1, 51) = 66.80, *MSE* = 2,540.31, *p* < .001, ɳ²_p_ = .57, reflected shorter RTs with the compatible mapping (*M* = 487 ms, *SD* = 63) than with the incompatible mapping (*M* = 544 ms, *SD* = 91). Importantly, however, and similar to the analysis including outlier participants according to Tukey, the two-way interaction was non-significant, *F*(1, 51) = 1.24, *MSE* = 1,277.27, *p* = .271, ɳ²_p_ = .02, revealing similar mapping effects in the two tasks.

Pairwise comparison revealed that, in the size-location task, RTs were significantly shorter with the compatible than with the incompatible mapping, *t*(51) = 8.82, *p* < .001, *d* = 1.22, BF_+0_ > 10,000.00, reflecting a regular SSARC effect of 63 ms (cf. Figure 2) and extreme evidence for its presence. In the location-size task, RTs were significantly shorter with the compatible than with the incompatible mapping, *t*(51) = 5.26, *p* < .001, *d* = 0.73, BF_+0_ = 5,978.89, revealing a reciprocal SSARC effect of 52 ms (cf. Figure 2) and extreme evidence for its presence.

In the analysis of error percentages without outliers, the two-factorial ANOVA with *Task* and *Mapping* as within-subjects factors again revealed a significant main effect of *Mapping*, *F*(1, 51) = 54.42, *MSE* = 8.93, *p* < .001, ɳ²_p_ = .52, indicating less errors with the compatible mapping (*M* = 1.27, *SD* = 1.73) than with the incompatible mapping (*M* = 4.33, *SD* = 3.82). A non-significant main effect of *Task*, *F*(1, 51) = 0.13, *MSE* = 4.00, *p* = .719, ɳ²_p_ < .01, indicated similar error percentages in the size-location (*M* = 2.75, *SD* = 3.20) and the location-size task (*M* = 2.85, *SD* = 3.47). However, after the exclusion of outliers, the formerly significant *Task* x *Mapping* interaction became non-significant, *F*(51) = 0.83, *MSE* = 5.07, *p* = .366, ɳ²_p_ = 0.02, indicating similar mapping effects between the two tasks when outlier datasets were excluded. This can be attributed to the observation that, by removing outliers, the reciprocal SSARC effect in error percentages increased from 2.13% (*d* = 0.42) to 2.77% (*d* = 0.69) while evidence for its presence increased from strong (BF_+0_ = 14.08) to extreme (BF_+0_ = 2,533.26).

The exclusion of outliers did not affect the pattern of results in the distributional analysis. Both two-way interactions *Quartile* x *Task* and *Quartile* x *Mapping* were again significant. The significant *Task* × *Quartile* interaction, *F*(3, 153) = 6.57, *MSE* = 625.31, *p* < .001, ɳ²_p_ = .11, reflected that, with increasing RTs, mean RTs in the location-size task became increasingly slower compared to the size-location task. The range of RTs was thus smaller in the size-location task compared to the location-size task. The significant *Mapping* × *Quartile* interaction, *F*(3, 153) = 54.26, *MSE* = 1,223.55, *p* < .001, ɳ²_p_ = .52, reflected that mapping effects increased with increasing RTs. Crucially, and similar to the analysis including outlier participants according to Tukey, the three-way interaction, *F*(3, 153) = 1.43, *MSE* = 811.74, *p* = .236, ɳ²_p_ = .03, was non-significant revealing similar time courses of the mapping effects between both tasks.

In the size-location task, the regular SSARC effect increased from 29 ms in the first quartile to 44 ms in the second quartile, 58 ms in the third quartile and 121 ms in the fourth quartile. Post-hoc tests comparing the compatible and incompatible mapping condition for each quartile revealed that the regular SSARC effect was significant in all four quartiles, all *t*s ≥ 6.10, all *p*s*_Tukey_* < .001. In the location-size task, the reciprocal SSARC effect increased from 25 ms in the first quartile to 36 ms in the second quartile, 51 ms in the third quartile and 96 ms in the fourth quartile. Post-hoc tests indicated that the reciprocal SSARC effect was significant in all four quartiles, all *t*s ≥ 3.99, all *p*s*_Tukey_* ≤ .017.
